# Supplementary material for: Metabolic Adjustments following Glutaminase Inhibition by CB-839 in Glioblastoma Cell Lines
Source: Cancers (Basel). 2023 Jan 15;15(2):531. doi: 10.3390/cancers15020531 (PMC9856342; doi:10.3390/cancers15020531)
Supplement: Supplementary file 1 [file cancers-15-00531-s001.zip › cancers-2134575-SI.pdf]

Supplementary Table S1. Significantly altered metabolites in T98G cell line treated with CB-839 1 μM. Abundances are provided as the normalized abundance mean of triplicates. Fold change is calculated by dividing mean abundance of the treated samples by the mean abundance of the control sample. The total ion count of the sample was used for normalizing each metabolite abundance.

**Compound Name**	**T98G** (Normalized metabolite abundance)	SD	**T98G +CB-839 1 μM** (Normalized metabolite abundance)	SD	Fold Change (T98G +CB-839 1 μM/T98G)	p-value (Student's T Test)
1-METHYLADENOSINE	1,658E-05	5,396E-06	5,747E-05	6,818E-06	3,466	0,00124
1-METHYL-L-HISTIDINE	1,672E-04	1,703E-05	2,059E-04	1,743E-06	1,231	0,01741
2-AMINOPHENOL-2	1,371E-05	1,893E-06	8,449E-06	1,057E-06	0,616	0,01366
2-ETHYL-2-HYDROXYBUTYRIC ACID	4,000E-06	5,810E-07	2,261E-06	8,998E-07	0,565	0,04823
2-HYDROXYBUTYRATE	4,961E-05	4,744E-06	3,199E-05	7,723E-06	0,645	0,02814
2-HYDROXYGLUTARIC	4,735E-04	1,589E-05	1,023E-04	1,062E-05	0,216	0,00000
3-(2-HYDROXYPHENYL)PROPANOATE	1,802E-05	6,758E-06	3,889E-05	2,475E-06	2,158	0,00738
3,4-DIHYDROXYHYDROCINNAMIC ACID	3,228E-04	6,436E-06	1,089E-03	3,663E-05	3,375	0,00000
3-HYDROXYBENZALDEHYDE	3,109E-05	7,152E-06	6,372E-05	4,677E-06	2,050	0,00271
3-METHOXYTYROSINE	1,037E-05	1,226E-06	1,506E-05	1,734E-06	1,452	0,01869
3-METHYL-2-OXOVALERATE	5,072E-04	8,511E-05	1,971E-04	3,010E-05	0,389	0,00401
3-METHYLADENINE-3	7,098E-05	4,754E-06	1,905E-04	1,964E-05	2,685	0,00051
4-ACETAMIDOBUTANOATE	5,473E-05	4,084E-06	8,970E-05	6,129E-06	1,639	0,00119
4-GUANIDINOBUTANOATE-2	2,123E-04	7,568E-06	1,298E-04	6,497E-06	0,611	0,00014
4-HYDROXYPHENYL-2-PROPIONIC ACID	1,762E-05	6,116E-06	3,780E-05	1,487E-06	2,145	0,00515
5-AMINOPENTANOATE	2,783E-03	3,945E-04	4,473E-03	2,194E-04	1,607	0,00292
5-AMINOPENTANOIC ACID	6,019E-05	1,874E-06	8,908E-06	9,847E-07	0,148	0,00000
5-HYDROXYTRYPTOPHAN	3,519E-05	1,344E-06	3,871E-05	1,502E-06	1,100	0,03899
5-METHYLCYTOSINE	1,239E-05	6,635E-07	2,740E-04	4,157E-05	22,113	0,00040
5-METHYLURIDINE	2,217E-05	2,650E-06	1,670E-05	1,209E-06	0,753	0,03131
6-HYDROXYDOPAMINE	1,626E-05	3,057E-06	7,881E-05	3,386E-06	4,847	0,00002
7-HYDROXYOCTANOATE	1,284E-06	1,075E-06	4,514E-06	1,087E-06	3,515	0,02157
ACETOACETATE	3,433E-05	7,813E-07	5,796E-06	2,112E-07	0,169	0,00000
AICAR	1,507E-05	9,261E-06	1,457E-04	1,362E-05	9,671	0,00016
ALPHA-KETOISOVALERATE	9,181E-05	1,041E-05	4,625E-05	4,708E-06	0,504	0,00230
AMINOADIPATE	9,535E-04	1,137E-04	1,363E-04	7,750E-06	0,143	0,00024
ARGININOSUCCINATE	7,871E-05	6,125E-06	7,770E-06	1,259E-06	0,099	0,00004
ASPARTATE	6,427E-04	1,189E-04	2,929E-05	2,205E-06	0,046	0,00087
BETAINE	4,209E-03	4,468E-04	1,005E-02	3,490E-04	2,388	0,00006
BUTYRYLCARNITINE	1,504E-02	5,498E-04	5,474E-03	9,712E-05	0,364	0,00001
CDP-ETHANOLAMINE	2,297E-03	1,680E-04	1,889E-03	7,672E-05	0,822	0,01858
CIS-ACONITATE-1	2,298E-04	8,716E-06	2,509E-05	4,617E-06	0,109	0,00000

CIS-ACONITATE-2	1,029E-04	1,513E-06	3,988E-05	2,499E-06	0,388	0,00000
CITRATE	1,246E-02	6,716E-04	1,262E-03	7,546E-05	0,101	0,00001
CITRULLINE	5,343E-05	4,878E-06	9,968E-05	4,097E-06	1,865	0,00023
CREATINE	2,703E-02	6,798E-04	3,100E-02	5,150E-04	1,147	0,00128
CYCLIC AMP	1,365E-04	6,340E-06	1,093E-04	5,195E-06	0,800	0,00452
CYCLIC GMP	1,151E-04	1,147E-05	8,208E-05	8,289E-06	0,713	0,01555
DECADIENOYLCARNITINE-2	1,208E-04	6,959E-06	1,542E-04	1,639E-05	1,277	0,03139
DECENOYLCARNITINE	8,520E-06	6,212E-07	2,721E-06	1,232E-06	0,319	0,00189
DEOXYADENOSINE MONOPHOSPHATE	2,792E-05	4,163E-06	1,108E-05	3,298E-06	0,397	0,00536
DEOXYCYTIDINE-1	4,160E-06	2,748E-06	2,216E-05	3,530E-06	5,328	0,00223
DEOXYCYTIDINE-DIPHOSPHATE	1,439E-05	2,321E-06	8,914E-06	1,797E-06	0,619	0,03188
DEOXYGUANOSINE-MONOPHOSPHATE	1,409E-03	4,804E-04	5,038E-04	2,542E-05	0,358	0,03110
D-GLUCURONOLACTONE	7,075E-05	5,445E-06	1,250E-04	5,999E-06	1,767	0,00032
DL-2-AMINOCTANOATE	1,349E-05	4,437E-07	2,980E-05	1,625E-06	2,209	0,00007
DOPAMINE-3	1,681E-05	4,778E-07	8,255E-06	6,077E-07	0,491	0,00004
D-RIBOSE 5-PHOSPHATE	1,028E-04	6,933E-06	1,666E-04	1,154E-05	1,620	0,00120
ETHANOLAMINE PHOSPHATE	2,356E-05	9,219E-07	6,518E-05	2,152E-06	2,766	0,00001
FRUCTOSE 1,6-BISPHOSPHATE	1,048E-04	2,021E-05	1,852E-04	2,338E-05	1,768	0,01073
FRUCTOSE 6-PHOSPHATE	3,999E-04	1,604E-05	8,718E-04	6,141E-05	2,180	0,00021
FUMARATE	1,369E-04	1,131E-05	2,227E-06	1,644E-06	0,016	0,00054
GLUCONOLACTONE	7,117E-06	1,813E-06	1,452E-05	2,493E-06	2,040	0,01416
GLUTAMATE	4,411E-02	3,040E-03	7,231E-03	5,489E-04	0,164	0,00003
GLUTAMINE	9,000E-03	9,363E-04	1,530E-02	6,939E-04	1,700	0,00072
GLUTATHIONE REDUCED	1,542E-01	6,161E-03	1,243E-01	2,216E-03	0,806	0,00138
GLYCERALDEHYDE 3-PHOSPHATE	2,255E-04	2,170E-05	1,652E-04	6,111E-06	0,732	0,00976
GLYCERATE	4,441E-05	6,668E-06	1,528E-05	2,180E-06	0,344	0,00198
GLYCEROL 3-PHOSPHATE	7,803E-05	1,028E-05	1,238E-04	9,691E-06	1,586	0,00496
GLYCINE	1,873E-05	1,622E-06	4,229E-05	9,155E-06	2,257	0,01180
GUANINE-2	6,512E-04	4,562E-05	7,953E-04	3,384E-05	1,221	0,01174
HEXANOYLCARNITINE	5,066E-05	6,088E-06	2,182E-05	8,199E-06	0,431	0,00810
HISTIDINE	3,181E-04	1,413E-05	3,898E-04	9,041E-06	1,225	0,00178
HOMOCYSTEINE THIOLACTONE	2,199E-05	3,213E-06	3,051E-05	3,532E-06	1,388	0,03650
HOMOSERINE	3,500E-04	2,755E-05	5,369E-04	3,398E-05	1,534	0,00178
HYDROQUINONE	1,263E-05	1,115E-06	8,113E-06	3,775E-07	0,643	0,00267
HYDROXYPHENYLLACTATE	3,225E-04	6,224E-06	1,089E-03	3,632E-05	3,376	0,00000
HYPOXANTHINE	6,582E-04	3,000E-04	4,354E-03	7,411E-05	6,616	0,00003
INDOLEACETALDEHYDE	3,577E-06	1,317E-06	6,371E-06	9,102E-07	1,781	0,03905
INOSINE MONOPHOSPHATE	4,384E-04	1,906E-05	6,959E-03	2,865E-04	15,874	0,00000
ISOCITRATE	3,486E-04	2,006E-05	7,720E-05	2,198E-05	0,221	0,00009
ISOLEUCINE	1,118E-02	2,387E-03	1,543E-02	8,244E-04	1,380	0,04352
ISOVALERYLCARNITINE	1,316E-02	4,365E-04	3,888E-03	2,202E-04	0,296	0,00001
KYNURENINE	4,562E-04	4,670E-05	5,774E-04	1,208E-05	1,266	0,01215

L-ALANINE	1,619E-04	3,026E-05	1,229E-05	8,211E-07	0,076	0,00102
L-CARNITINE	4,001E-02	1,147E-03	1,097E-02	5,009E-04	0,274	0,00000
LEUCINE	3,157E-02	5,731E-03	4,628E-02	5,638E-03	1,466	0,03394
MALATE	5,269E-03	1,176E-03	2,496E-04	3,015E-05	0,047	0,00179
METHIONINE SULFOXIDE	2,101E-05	1,431E-06	3,873E-05	3,384E-06	1,844	0,00112
METHIONINE-1	5,014E-03	1,365E-04	6,625E-03	5,849E-04	1,321	0,00969
METHIONINE-2	1,263E-02	1,006E-03	1,761E-02	1,250E-03	1,394	0,00579
METHYLTHIOADENOSINE	6,672E-03	2,910E-04	8,416E-03	7,107E-04	1,261	0,01705
N,N,N-TRIMETHYLLYSINE	1,975E-05	2,371E-06	4,287E-05	8,039E-06	2,171	0,00879
N1-ACETYLSPERMINE	1,218E-06	6,464E-07	3,173E-06	8,812E-07	2,604	0,03631
N-A-ACETYL-L-ARGININE	1,822E-05	4,054E-06	3,228E-05	5,432E-06	1,772	0,02289
N-ACETYLALANINE	2,691E-04	2,971E-05	2,026E-04	2,674E-05	0,753	0,04494
N-ACETYLASPARAGINE	1,555E-05	1,983E-06	5,267E-05	6,010E-06	3,386	0,00053
N-ACETYLASPARTATE	4,670E-03	1,455E-04	3,281E-03	1,113E-04	0,702	0,00019
N-ACETYLASPARTYLGLUTAMATE	5,940E-04	2,375E-05	4,473E-04	1,769E-05	0,753	0,00101
N-ACETYLGLUTAMATE	9,178E-04	2,805E-05	1,443E-03	5,271E-05	1,573	0,00011
N-ACETYLGLYCINE	3,791E-05	3,366E-06	8,547E-05	5,256E-06	2,255	0,00019
N-ACETYLMETHIONINE	7,901E-04	6,934E-05	6,582E-04	3,850E-05	0,833	0,04504
N-ACETYLNEURAMINATE	7,720E-04	7,457E-05	6,211E-04	5,070E-05	0,805	0,04419
N-ACETYLPHENYLALANINE	9,838E-06	8,748E-07	1,699E-05	2,148E-06	1,727	0,00591
N-ACETYLSERINE	2,205E-04	4,604E-05	6,146E-04	4,073E-05	2,787	0,00037
N-FORMYL-L-METHIONINE	6,883E-05	2,637E-06	8,585E-05	8,919E-06	1,247	0,03382
NICOTINAMIDE MONONUCLEOTIDE	9,565E-05	1,941E-05	1,336E-04	1,313E-05	1,397	0,04849
N-METHYLGLUTAMATE	1,312E-03	1,798E-04	2,148E-04	7,634E-06	0,164	0,00046
O-ACETYLCARNITINE	1,935E-03	1,134E-04	2,200E-02	6,218E-05	11,372	0,00000
O-ACETYLCARNITINE-2	8,652E-03	5,022E-04	9,909E-02	1,461E-03	11,453	0,00000
O-ACETYLSERINE	1,510E-02	1,877E-03	2,225E-03	5,345E-05	0,147	0,00029
O-SUCCINYL-HOMOSERINE-1	4,497E-05	3,115E-06	1,099E-05	2,960E-06	0,244	0,00016
OXOGLUTARATE	6,531E-05	9,362E-06	4,894E-06	2,590E-06	0,075	0,00042
PHENYLALANINE	9,318E-02	7,782E-03	1,123E-01	4,278E-03	1,205	0,02046
PHENYLLACTATE	1,794E-05	6,430E-06	3,884E-05	2,761E-06	2,165	0,00663
PHOSPHORYLCHOLINE	1,266E-02	6,604E-04	1,950E-02	1,599E-03	1,540	0,00238
PHOSPHOSERINE	1,536E-05	2,252E-06	4,212E-06	4,613E-07	0,274	0,00110
PIPECOLATE	3,420E-04	1,246E-05	8,811E-04	3,120E-05	2,576	0,00001
P-OCTOPAMINE	1,457E-06	7,002E-07	3,576E-06	4,659E-07	2,455	0,01202
PORPHOBILINOGEN-2	2,185E-05	2,233E-06	8,799E-06	6,865E-07	0,403	0,00064
PROLINE	6,248E-03	7,396E-04	4,041E-03	1,401E-04	0,647	0,00708
PROPIONYLCARNITINE	3,150E-02	8,314E-04	2,005E-02	1,247E-03	0,636	0,00019
PYRIDOXAL-PHOSPHATE	4,790E-05	6,219E-06	6,514E-05	6,693E-06	1,360	0,03082
PYRIDOXINE-1	1,173E-04	6,840E-06	8,920E-05	3,844E-07	0,761	0,00209
PYRIDOXINE-2	1,203E-04	3,056E-06	8,753E-05	9,721E-07	0,728	0,00006
PYROGLUTAMATE	1,749E-03	1,117E-04	1,332E-03	1,279E-04	0,762	0,01313
PYRUVATE	2,288E-05	3,929E-06	1,026E-05	3,081E-06	0,448	0,01189
SARCOSINE	4,451E-04	1,830E-05	5,142E-04	3,337E-06	1,155	0,00301

SEDOHEPTULOSE-7-PHOSPHATE	5,304E-05	6,967E-06	2,301E-04	2,519E-05	4,337	0,00030
SPERMIDINE	1,960E-02	2,409E-03	2,456E-02	7,322E-04	1,253	0,02697
SPERMINE	7,077E-03	1,353E-03	4,555E-03	3,211E-04	0,644	0,03479
SUCCINATE	6,391E-04	8,647E-05	4,075E-04	8,638E-05	0,638	0,03045
THREONINE	2,605E-04	6,345E-05	3,761E-04	2,781E-05	1,444	0,04455
THYMIDINE-MONOPHOSPHATE	4,339E-04	1,010E-04	2,231E-04	6,699E-05	0,514	0,03942
TRANS-ACONITATE	5,971E-05	6,782E-06	1,610E-05	5,903E-06	0,270	0,00110
TRIGONELLINE	3,416E-04	1,159E-05	2,128E-04	1,630E-05	0,623	0,00037
TRIMETHYLAMINE	1,358E-05	2,285E-06	4,555E-06	6,643E-07	0,335	0,00278
TRYPTOPHAN	8,823E-03	1,640E-04	1,269E-02	6,387E-04	1,438	0,00053
TYRAMINE	9,090E-06	4,349E-06	1,829E-05	5,618E-07	2,012	0,02209
TYROSINE	2,085E-02	1,268E-03	3,167E-02	2,174E-03	1,519	0,00174
URACIL-1	4,213E-05	1,451E-07	2,073E-05	2,652E-06	0,492	0,00015
URIDINE MONOPHOSPHATE	2,648E-03	9,980E-05	1,273E-03	2,862E-04	0,481	0,00142
VALINE	2,664E-04	2,806E-05	5,466E-04	3,920E-05	2,052	0,00055

Supplementary Table S2. Significantly altered metabolites in LN229 cell line treated with CB-839 1 μM. Abundances are provided as the normalized abundance mean of triplicates. Fold change is calculated by dividing mean abundance of the treated samples by the mean abundance of the control sample. The total ion count of the sample was used for normalizing each metabolite abundance.

Compound Name	LN229	SD	LN229 +CB-839 1 μM	SD	Fold Change (LN229 +CB-839 1 μM/LN229)	p-value (Student's T Test)
1-AMINOCYCLOPROPANECARBOXYLATE	1,500E-05	2,080E-06	2,050E-06	6,530E-07	0,137	0,00000
2-ETHYL-2-HYDROXYBUTYRIC ACID	9,210E-06	1,250E-06	6,930E-06	1,850E-06	0,753	0,03180
2-HYDROXYGLUTARIC	2,020E-04	2,490E-05	1,600E-04	2,790E-05	0,792	0,02060
3-(2-HYDROXYPHENYL)PROPANOATE	8,610E-06	1,980E-06	2,180E-05	3,240E-06	2,529	0,00001
3,4-DIHYDROXYHYDROCINNAMIC ACID	2,910E-04	4,010E-05	1,910E-03	2,050E-04	6,549	0,00000
3,4-DIHYDROXYPHENYLACETATE-1	9,080E-06	2,460E-06	1,300E-05	1,940E-06	1,433	0,01175
3,4-DIHYDROXYPHENYLACETATE-2	1,630E-05	2,900E-06	2,310E-05	5,910E-06	1,418	0,02960
3-AMINO-5-HYDROXYBENZOATE	8,440E-06	3,510E-06	2,670E-06	2,070E-06	0,316	0,00606
3-HYDROXYOCTANOIC ACID	1,360E-05	2,780E-06	8,610E-06	1,020E-06	0,635	0,00218
3-METHOXYTYROSINE	5,400E-06	8,270E-07	6,740E-06	9,180E-07	1,247	0,02458
3-METHYLADENINE-3	7,730E-05	1,760E-05	2,000E-04	1,090E-05	2,584	0,00000
3-PHOSPHOGLYCERATE	2,870E-05	7,260E-06	1,820E-05	2,850E-06	0,636	0,00832
3-SULFINOALANINE	1,840E-05	3,640E-06	1,190E-05	1,280E-06	0,647	0,00211
4-ACETAMIDOBUTANOATE	3,920E-05	8,520E-06	7,860E-05	5,410E-06	2,006	0,00000
4-GUANIDINOBUTANOATE	6,300E-05	6,640E-06	4,380E-05	2,900E-06	0,695	0,00007
4-GUANIDINOBUTANOATE-2	2,530E-04	3,610E-05	2,060E-04	1,100E-05	0,816	0,01275
4-HYDROXYPHENYL-2-PROPIONIC ACID	7,500E-06	2,490E-06	2,120E-05	3,130E-06	2,826	0,00001
5-AMINOPENTANOATE	4,480E-03	1,280E-03	6,300E-03	1,350E-03	1,405	0,03809
5-AMINOPENTANOIC ACID	1,130E-05	1,980E-06	5,500E-06	1,700E-06	0,488	0,00030
6-HYDROXYDOPAMINE	1,860E-05	3,430E-06	3,080E-05	4,970E-06	1,653	0,00059
6-PHOSPHONOGLUCONO-D-LACTONE	1,090E-05	6,260E-06	4,170E-06	1,620E-06	0,382	0,03025
8-HYDROXY-DEOXYGUANOSINE	3,100E-04	3,710E-05	5,400E-04	1,010E-04	1,743	0,00038
ACETOACETATE	1,110E-05	3,120E-06	7,230E-06	2,260E-06	0,651	0,03356
ACETOIN	2,060E-05	4,000E-06	2,960E-05	6,920E-06	1,435	0,02050
ALPHA-KETOISOVALERATE	2,350E-05	4,750E-06	3,070E-05	5,850E-06	1,306	0,04141
AMINOADIPATE	1,690E-04	2,740E-05	6,640E-05	4,850E-06	0,392	0,00000
ARGININE	4,990E-04	9,320E-05	6,670E-04	1,130E-04	1,337	0,01828
ARGININOSUCCINATE	5,640E-05	8,280E-06	3,530E-05	9,690E-06	0,625	0,00228
ASPARTATE	7,590E-04	1,020E-04	4,220E-05	4,930E-06	0,056	0,00000
BETAINE	1,520E-03	3,700E-04	3,200E-03	5,470E-04	2,099	0,00010
BIS(2-ETHYLHEXYL)PHTHALATE	9,240E-05	4,870E-05	2,630E-04	1,790E-04	2,840	0,04839
BUTYRYLCARNITINE	1,850E-02	1,910E-03	1,500E-02	2,090E-03	0,813	0,01332
CHENODEOXYCHOLIC ACID	1,350E-05	1,130E-05	1,120E-04	1,060E-04	8,326	0,04714
CIS-ACONITATE-1	1,700E-04	5,820E-05	1,130E-04	2,090E-05	0,666	0,04758
CIS-ACONITATE-2	5,030E-05	1,910E-05	2,750E-05	3,750E-06	0,547	0,01677
CITRATE	1,020E-02	3,580E-03	6,020E-03	1,210E-03	0,592	0,02246
CITRULLINE	5,280E-05	6,880E-06	6,990E-05	6,750E-06	1,324	0,00146
CYCLIC AMP	1,650E-04	3,860E-05	1,040E-04	1,370E-05	0,627	0,00420
CYSTATHIONINE	8,130E-06	1,580E-06	6,310E-06	7,030E-07	0,776	0,02777

CYSTEINE	6,580E-06	2,040E-06	1,560E-05	5,300E-06	2,377	0,00292
DECANOYLCARNITINE	8,200E-05	8,910E-06	7,030E-05	5,520E-06	0,858	0,02127
DEOXYCARNITINE	9,760E-03	1,010E-03	8,050E-03	3,340E-04	0,824	0,00267
DEOXYCHOLATE	1,350E-05	1,130E-05	1,120E-04	1,070E-04	8,307	0,04793
DEOXYCHOLIC ACID	1,360E-05	1,130E-05	1,120E-04	1,070E-04	8,256	0,04790
DEOXYCYTIDINE-1	2,170E-06	5,860E-07	7,050E-06	2,690E-06	3,247	0,00146
DEOXYURIDINE	2,010E-06	7,190E-07	3,400E-06	1,120E-06	1,693	0,02855
D-GLUCURONOLACTONE	3,870E-05	1,040E-05	5,960E-05	8,620E-06	1,542	0,00344
DL-2-AMINOCTANOATE	1,370E-05	4,140E-06	2,030E-05	4,260E-06	1,489	0,02043
D-RIBOSE 5-PHOSPHATE	3,430E-05	8,940E-06	4,880E-05	6,720E-06	1,421	0,01011
ETHANOLAMINE PHOSPHATE	8,870E-06	1,890E-06	1,870E-05	1,510E-06	2,104	0,00000
FRUCTOSE 1,6-BISPHOSPHATE	3,620E-05	3,710E-06	8,460E-05	1,360E-05	2,334	0,00001
FRUCTOSE 6-PHOSPHATE	1,900E-04	2,050E-05	2,670E-04	2,760E-05	1,410	0,00025
FUMARATE	1,240E-04	3,010E-05	2,170E-05	6,840E-06	0,176	0,00001
GLUTAMATE	2,660E-02	3,270E-03	7,320E-03	7,290E-04	0,275	0,00000
GLUTAMINE	8,270E-03	1,060E-03	1,180E-02	1,120E-03	1,422	0,00024
GLUTARATE	6,300E-05	1,050E-05	9,000E-05	1,710E-05	1,428	0,00804
GLUTATHIONE REDUCED	9,070E-02	1,110E-02	6,710E-02	9,630E-03	0,740	0,00281
GLYCINE	1,020E-05	1,890E-06	1,930E-05	3,560E-06	1,897	0,00025
GUANINE-1	5,130E-04	1,430E-04	9,770E-04	1,900E-04	1,904	0,00075
GUANINE-2	8,680E-04	2,180E-04	1,110E-03	1,340E-04	1,274	0,04607
GUANOSINE	1,620E-04	3,910E-05	3,140E-04	8,770E-05	1,944	0,00298
GUANOSINE MONOPHOSPHATE	9,690E-04	1,580E-04	7,310E-04	1,530E-04	0,755	0,02446
HEXOSE	1,260E-04	1,800E-05	6,260E-05	1,730E-05	0,498	0,00010
HOMOCYSTEINE THIOLACTONE	1,820E-05	1,330E-06	2,260E-05	1,610E-06	1,248	0,00036
HOMOSERINE	4,380E-04	7,770E-05	5,640E-04	6,150E-05	1,288	0,01087
HYDROXYKYNURENINE	1,640E-05	3,780E-06	1,260E-05	1,020E-06	0,770	0,03982
HYDROXYPHENYLLACTATE	2,900E-04	4,010E-05	1,910E-03	2,050E-04	6,572	0,00000
HYPOXANTHINE	3,700E-04	1,270E-04	7,920E-04	1,680E-04	2,141	0,00063
INDOLEACETALDEHYDE	2,010E-06	6,360E-07	9,100E-06	2,280E-06	4,537	0,00003
INOSINE	2,700E-04	5,930E-05	6,460E-04	2,580E-04	2,392	0,00598
INOSINE MONOPHOSPHATE	2,220E-04	4,120E-05	4,810E-04	4,620E-05	2,170	0,00000
ISOLEUCINE	1,730E-02	4,090E-03	2,330E-02	2,680E-03	1,349	0,01279
KYNURENINE	2,820E-04	3,280E-05	3,360E-04	2,370E-05	1,191	0,00860
L-ALANINE	1,620E-04	1,590E-05	9,150E-06	2,230E-06	0,057	0,00000
L-CARNITINE	3,640E-02	2,490E-03	2,620E-02	1,050E-03	0,719	0,00000
LEUCINE	4,310E-02	7,680E-03	5,770E-02	7,850E-03	1,338	0,00866
L-ORNITHINE	6,770E-05	9,010E-06	9,580E-05	1,040E-05	1,416	0,00053
LYSINE	1,160E-04	1,150E-05	1,450E-04	1,820E-05	1,249	0,00818
MALATE	4,230E-03	5,770E-04	8,940E-04	1,810E-04	0,212	0,00000
METHIONINE SULFOXIDE	2,230E-05	2,520E-06	3,560E-05	4,320E-06	1,598	0,00007
METHIONINE-1	7,320E-03	1,260E-03	9,710E-03	1,020E-03	1,327	0,00473
METHIONINE-2	1,840E-02	3,070E-03	2,720E-02	3,430E-03	1,480	0,00085
MEVALONATE	4,320E-06	8,720E-07	2,970E-06	8,330E-07	0,689	0,02144
N,N,N-TRIMETHYLLYSINE	1,150E-05	2,760E-06	1,620E-05	3,400E-06	1,406	0,02556
N-ACETYLASPARAGINE	8,640E-06	1,250E-06	2,550E-05	4,570E-06	2,957	0,00001

N-ACETYLASPARTATE	1,840E-03	3,450E-04	1,320E-03	1,850E-04	0,720	0,00914
N-ACETYLGLUTAMATE	6,960E-04	1,550E-04	1,310E-03	6,920E-05	1,882	0,00000
N-ACETYLGLYCINE	2,470E-05	3,060E-06	4,480E-05	9,190E-06	1,814	0,00048
N-ACETYLNEURAMINATE	1,130E-03	1,780E-04	5,570E-04	4,220E-05	0,492	0,00002
N-ACETYLPUTRESCINE	5,140E-05	1,640E-05	3,090E-04	9,990E-05	6,001	0,00010
N-ACETYLSERINE	1,180E-04	1,980E-05	2,850E-04	5,450E-05	2,412	0,00004
N-METHYLGLUTAMATE	3,190E-04	2,480E-05	1,260E-04	6,010E-06	0,396	0,00000
O-ACETYLCARNITINE	2,110E-03	1,930E-04	4,230E-03	6,070E-04	2,006	0,00001
O-ACETYLCARNITINE-2	9,680E-03	7,350E-04	2,180E-02	3,260E-03	2,255	0,00000
O-ACETYLSERINE	1,350E-02	1,610E-03	3,100E-03	1,930E-04	0,230	0,00000
O-SUCCINYL-HOMOSERINE-1	3,750E-05	6,180E-06	1,560E-05	2,040E-06	0,416	0,00001
OXOGLUTARATE	2,230E-05	4,470E-06	7,010E-06	3,080E-06	0,314	0,00004
PALMITOYLCARNITINE	7,480E-05	2,740E-05	1,330E-04	3,900E-05	1,771	0,01415
PHENYLALANINE	8,760E-02	7,660E-03	1,030E-01	6,360E-03	1,175	0,00359
PHENYLETHANOLAMINE	2,640E-04	2,970E-05	3,160E-04	3,400E-05	1,198	0,01785
PHENYLLACTATE	8,430E-06	1,920E-06	2,160E-05	3,160E-06	2,564	0,00001
PHOSPHOCREATINE	1,080E-04	3,610E-05	4,520E-05	2,020E-05	0,419	0,00404
PHOSPHORYLCHOLINE	2,900E-02	3,470E-03	3,690E-02	5,880E-03	1,274	0,01740
PHOSPHOSERINE	1,210E-05	2,480E-06	3,040E-06	6,360E-07	0,251	0,00001
PIPECOLATE	9,810E-05	1,130E-05	4,970E-04	7,650E-05	5,066	0,00000
PORPHOBILINOGEN-2	3,010E-05	5,050E-06	2,190E-05	2,660E-06	0,727	0,00540
PROLINE	8,280E-03	2,110E-03	1,190E-03	4,480E-04	0,144	0,00001
PROPIONYLCHOLINE	9,410E-05	1,970E-05	5,690E-05	1,350E-05	0,604	0,00341
PUTRESCINE-NH3	1,840E-04	2,570E-05	2,380E-04	1,670E-05	1,299	0,00137
PYROGLUTAMATE	2,200E-03	2,960E-04	2,910E-03	4,990E-04	1,319	0,01417
PYRUVATE	1,910E-05	3,980E-06	7,730E-06	1,590E-06	0,405	0,00007
S-ADENOSYLHOMOCYSTEINE-1	2,370E-04	4,850E-05	1,470E-04	5,920E-05	0,622	0,01688
SALSOLINOL-1	5,530E-04	7,120E-05	4,440E-04	4,770E-05	0,802	0,01052
SALSOLINOL-2	1,750E-03	1,600E-04	1,450E-03	1,760E-04	0,829	0,01171
SEDOHEPTULOSE-7-PHOSPHATE	1,050E-05	4,030E-06	3,720E-05	6,720E-06	3,558	0,00001
SPERMIDINE	1,720E-02	2,990E-03	2,270E-02	2,110E-03	1,315	0,00458
SUCCINATE	8,860E-04	2,050E-04	4,730E-04	9,630E-05	0,534	0,00122
TAURINE	2,380E-03	4,830E-04	1,170E-03	1,510E-04	0,492	0,00016
THYMIDINE-MONOPHOSPHATE	1,070E-04	1,790E-05	6,530E-05	1,910E-05	0,609	0,00285
TRANS-2-OCTENOYL-L-CARNITINE	1,170E-03	1,250E-04	9,490E-04	7,220E-05	0,808	0,00336
TRIGONELLINE	4,020E-04	1,050E-04	1,330E-04	8,350E-06	0,331	0,00009
TRIMETHYLAMINE	1,340E-05	2,490E-06	1,000E-05	2,680E-06	0,745	0,04445
TRYPTOPHAN	7,790E-03	1,180E-03	1,140E-02	8,400E-04	1,465	0,00011
TYRAMINE	1,470E-05	2,920E-06	1,920E-05	2,780E-06	1,303	0,02181
TYROSINE	2,280E-02	3,620E-03	3,370E-02	3,210E-03	1,477	0,00026
URACIL-1	3,500E-05	1,290E-05	2,160E-05	3,650E-06	0,617	0,03359
URIDINE MONOPHOSPHATE	2,330E-03	1,140E-03	1,040E-03	7,500E-04	0,445	0,04227
VALINE	3,590E-04	3,980E-05	4,900E-04	7,750E-05	1,366	0,00417
XANTHOSINE	8,920E-05	1,400E-05	1,110E-04	1,290E-05	1,241	0,01995

Supplementary Table S3. Significantly altered metabolites in U87MG cell line treated with CB-839 1 μM. Abundances are provided as the normalized abundance mean of triplicates. Fold change is calculated by dividing mean abundance of the treated samples by the mean abundance of the control sample. The total ion count of the sample was used for normalizing each metabolite abundance.

Compound Name	U87MG	SD	U87MG +CB-839 1μM	SD	Fold Change (U87MG +CB-839 1 μM/U87MG)	p-value (Student's T Test)
1-AMINOCYCLOPROPANECARBOXYLATE	2,318E-05	5,545E-07	5,077E-06	1,955E-06	0,219	0,00010
1-METHYL-L-HISTIDINE	3,648E-05	1,839E-07	4,300E-05	3,669E-06	1,179	0,03712
2-HYDROXYGLUTARIC	3,422E-04	3,183E-05	2,175E-04	6,629E-06	0,636	0,00266
3-(2-HYDROXYPHENYL)PROPANOATE	1,294E-05	1,497E-06	1,775E-05	2,084E-06	1,372	0,03144
3,4-DIHYDROXYHYDROCINNAMIC ACID	2,310E-04	7,982E-06	5,984E-04	1,507E-05	2,590	0,00000
3-AMINO-5-HYDROXYBENZOATE	1,879E-06	1,971E-07	6,365E-06	1,176E-06	3,387	0,00286
3-HYDROXYBENZALDEHYDE	4,180E-06	6,194E-07	8,727E-06	1,785E-06	2,088	0,04509
3-METHYLADENINE-3	7,221E-05	7,652E-06	1,781E-04	2,537E-05	2,466	0,00229
4-ACETAMIDOBUTANOATE	2,690E-04	2,640E-07	3,995E-04	1,169E-05	1,485	0,00004
4-HYDROXYPHENYL-2-PROPIONIC ACID	1,106E-05	2,632E-06	1,626E-05	1,176E-06	1,470	0,03539
5-AMINOPENTANOIC ACID	8,670E-05	8,918E-06	7,067E-05	2,195E-06	0,815	0,03901
6-HYDROXYDOPAMINE	4,407E-05	9,491E-06	7,504E-05	5,149E-06	1,703	0,00766
6-PHOSPHOGLUCONATE	6,057E-04	1,311E-04	3,015E-04	2,690E-05	0,498	0,01701
6-PHOSPHONOGLUCONO-D-LACTONE	3,806E-05	6,608E-06	1,485E-05	6,487E-07	0,390	0,00375
ACETOACETATE	1,896E-05	2,678E-06	1,235E-05	1,396E-06	0,651	0,01924
ADENOSINE	3,228E-03	3,429E-04	1,911E-03	3,216E-04	0,592	0,00831
AICAR	2,534E-06	6,207E-07	6,706E-06	9,360E-07	2,646	0,00300
ALPHA-KETOISOVALERATE	5,321E-05	2,133E-06	4,343E-05	3,915E-06	0,816	0,01910
AMINOADIPATE	1,351E-03	1,807E-04	9,727E-04	2,721E-05	0,720	0,02309
ARGININOSUCCINATE	2,649E-05	1,854E-06	9,790E-06	1,622E-06	0,370	0,00030
ASPARTATE	1,644E-04	2,712E-05	4,887E-05	9,896E-06	0,297	0,00227
BETAINE	1,347E-03	5,137E-04	2,787E-03	2,338E-04	2,070	0,01151
BUTYRYLCARNITINE	4,425E-03	1,968E-04	3,677E-03	1,444E-04	0,831	0,00608
CIS-ACONITATE-1	2,808E-04	3,137E-05	1,303E-04	2,512E-06	0,464	0,00116
CIS-ACONITATE-2	2,015E-04	1,023E-05	1,234E-04	4,628E-06	0,613	0,00027
CITRATE	1,664E-02	2,121E-03	7,227E-03	3,394E-04	0,434	0,00161
CITRULLINE	1,627E-05	3,243E-06	2,950E-05	2,507E-06	1,814	0,00502
DEOXYADENOSINE MONOPHOSPHATE	2,023E-05	8,570E-07	1,213E-05	2,543E-06	0,600	0,00640
DOPAMINE-3	2,722E-05	1,188E-06	2,383E-05	9,824E-07	0,876	0,01908
ETHANOLAMINE PHOSPHATE	2,089E-05	7,696E-07	3,343E-05	2,440E-06	1,600	0,00106
FUMARATE	4,011E-05	3,890E-06	1,280E-05	1,471E-06	0,319	0,00034
GLUCONATE	3,418E-04	1,357E-05	2,865E-04	1,170E-05	0,838	0,00592
GLUTAMATE	4,087E-02	1,784E-03	1,855E-02	2,671E-04	0,454	0,00003
GLUTAMINE	9,435E-03	4,081E-04	1,323E-02	8,613E-05	1,402	0,00009
GLUTARATE	9,936E-05	1,550E-06	1,281E-04	1,719E-06	1,290	0,00003
GLYCEROL 3-PHOSPHATE	2,277E-04	1,156E-05	3,095E-04	1,420E-05	1,359	0,00150
HOMOSERINE	3,322E-04	3,853E-06	4,327E-04	1,150E-05	1,303	0,00014
HYDROXYPHENYLLACTATE	2,304E-04	8,281E-06	5,984E-04	1,455E-05	2,597	0,00000

ISOCITRATE	6,011E-04	6,756E-05	3,176E-04	5,849E-06	0,528	0,00193
ISOLEUCINE	7,047E-03	7,716E-04	1,010E-02	1,719E-03	1,433	0,04852
ISOVALERYLCARNITINE	1,958E-02	1,842E-03	9,942E-03	3,538E-04	0,508	0,00088
KYNURENINE	7,456E-04	6,601E-05	9,847E-04	2,988E-05	1,321	0,00464
L-ALANINE	4,227E-05	6,571E-06	1,492E-05	1,492E-06	0,353	0,00215
L-CARNITINE	1,657E-02	1,171E-03	9,713E-03	7,499E-04	0,586	0,00103
MALATE	1,425E-03	2,340E-04	5,606E-04	5,265E-05	0,393	0,00336
METHIONINE SULFOXIDE	1,708E-05	1,779E-06	2,345E-05	1,988E-06	1,373	0,01443
N,N,N-TRIMETHYLLYSINE	2,293E-05	4,227E-06	3,205E-05	3,121E-06	1,398	0,03969
N-ACETYLALANINE	1,165E-04	7,328E-06	1,372E-04	3,122E-06	1,178	0,01077
N-ACETYLASPARAGINE	9,915E-06	1,954E-06	1,456E-05	1,792E-06	1,469	0,03853
N-ACETYLGLUTAMATE	4,431E-03	2,124E-04	6,547E-03	9,566E-05	1,477	0,00010
N-ACETYLGLYCINE	1,120E-05	1,678E-06	2,103E-05	1,255E-06	1,877	0,00125
N-ACETYLLEUCINE	2,052E-05	2,174E-06	2,532E-05	1,593E-06	1,234	0,03687
N-ACETYLNEURAMINATE	9,578E-04	3,646E-05	8,776E-04	2,484E-05	0,916	0,03450
N-ACETYLSERINE	4,934E-05	1,374E-05	1,178E-04	1,526E-05	2,387	0,00447
N-FORMYL-L-METHIONINE	1,021E-04	3,177E-06	1,202E-04	2,207E-06	1,178	0,00125
O-ACETYLCARNITINE	4,420E-03	6,630E-04	1,161E-02	1,334E-03	2,627	0,00112
O-ACETYLCARNITINE-2	2,141E-02	2,316E-03	4,674E-02	5,900E-04	2,183	0,00005
O-ACETYLSERINE	1,190E-02	1,323E-03	4,879E-03	2,583E-04	0,410	0,00084
O-SUCCINYL-HOMOSERINE-1	2,795E-05	3,860E-06	7,040E-06	3,532E-06	0,252	0,00229
OXOGLUTARATE	4,734E-05	4,801E-06	1,933E-05	1,883E-06	0,408	0,00071
PALMITOYLCARNITINE	2,274E-05	3,467E-06	3,161E-05	1,522E-06	1,390	0,01540
PANTOTHENATE	3,158E-02	1,271E-03	3,472E-02	7,280E-04	1,099	0,02060
PHENYLLACTATE	1,255E-05	1,252E-06	1,764E-05	1,833E-06	1,405	0,01655
PIPECOLATE	3,113E-04	5,695E-05	7,772E-04	1,973E-05	2,497	0,00018
PROPIONYLCARNITINE	2,757E-02	2,354E-03	2,098E-02	1,362E-04	0,761	0,00839
PTERIN	4,422E-06	1,181E-06	7,820E-06	1,377E-06	1,768	0,03153
RIBOFLAVIN	6,369E-06	8,186E-07	8,063E-06	6,313E-07	1,266	0,04689
SPERMINE	5,946E-03	3,788E-04	4,726E-03	2,789E-04	0,795	0,01088
SUBERATE	2,093E-04	9,870E-06	2,300E-04	8,102E-06	1,099	0,04816
SUCCINATE	4,305E-04	4,749E-05	3,136E-04	7,562E-06	0,729	0,01360
TRANS-ACONITATE	9,442E-05	6,004E-06	5,469E-05	8,025E-06	0,579	0,00236
TRYPTOPHAN	6,212E-03	1,676E-04	7,609E-03	8,517E-05	1,225	0,00021
TYROSINE	1,259E-02	2,588E-04	1,537E-02	3,092E-04	1,221	0,00028
VALINE	1,762E-04	1,502E-05	2,394E-04	1,993E-05	1,359	0,01183
XANTHOSINE	9,955E-05	5,489E-06	1,354E-04	1,367E-06	1,360	0,00039

Supplementary Table S4. Comparison of significantly altered metabolites among the three cell lines treated with CB-839 1 μM. Fold change is provided for each significantly altered metabolite. Since there are metabolites that significantly change for one cell line but not for another, "ns" is used for metabolites not significantly changing.

Compound Name	T98G+CB-839 1μM/T98G (Fold change)	LN229+CB-839 1μM/LN229 (Fold change)	U87MG+CB-839 1μM/U87MG (Fold change)
1-AMINOCYCLOPROPANECARBOXYLATE	ns	0,137	0,219
1-METHYLADENOSINE	3,466	ns	ns
1-METHYL-L-HISTIDINE	1,231	ns	1,179
2-AMINOPHENOL-2	0,616	ns	ns
2-ETHYL-2-HYDROXYBUTYRIC ACID	0,565	0,753	ns
2-HYDROXYBUTYRATE	0,645	ns	ns
2-HYDROXYGLUTARIC	0,216	0,792	0,636
3-(2-HYDROXYPHENYL)PROPANOATE	2,158	2,529	1,372
3,4-DIHYDROXYHYDROCINNAMIC ACID	3,375	6,549	2,590
3,4-DIHYDROXYPHENYLACETATE-1	ns	1,433	ns
3,4-DIHYDROXYPHENYLACETATE-2	ns	1,418	ns
3-AMINO-5-HYDROXYBENZOATE	ns	0,316	3,387
3-HYDROXYBENZALDEHYDE	2,050	ns	2,088
3-HYDROXYOCTANOIC ACID	ns	0,63516566	ns
3-METHOXYTYROSINE	1,452	1,247	ns
3-METHYL-2-OXOVALERATE	0,389	ns	ns
3-METHYLADENINE-3	2,685	2,584	2,466
3-PHOSPHOGLYCERATE	ns	0,636	ns
3-SULFINOALANINE	ns	0,647	ns
4-ACETAMIDOBUTANOATE	1,639	2,006	1,485
4-GUANIDINOBUTANOATE	ns	0,695	ns
4-GUANIDINOBUTANOATE-2	0,611	0,816	ns
4-HYDROXYPHENYL-2-PROPIONIC ACID	2,145	2,826	1,470
5-AMINOPENTANOATE	1,607	1,405	ns
5-AMINOPENTANOIC ACID	0,148	0,488	0,815
5-HYDROXYTRYPTOPHAN	1,100	ns	ns
5-METHYLCYTOSINE	22,113	ns	ns
5-METHYLURIDINE	0,753	ns	ns
6-HYDROXYDOPAMINE	4,847	1,653	1,703
6-PHOSPHOGLUCONATE	ns	ns	0,498
6-PHOSPHONOGLUCONO-D-LACTONE	ns	0,382	0,390
7-HYDROXYOCTANOATE	3,515	ns	ns
8-HYDROXY-DEOXYGUANOSINE	ns	1,743	ns
ACETOACETATE	0,169	0,651	0,651
ACETOIN	ns	1,435	ns
ADENOSINE	ns	ns	0,592
AICAR	9,671	ns	2,646
ALPHA-KETOISOVALERATE	0,504	1,306	0,816
AMINOADIPATE	0,143	0,392	0,720
ARGININE	ns	1,337	ns

ARGININOSUCCINATE	0,099	0,625	0,370
ASPARTATE	0,046	0,056	0,297
BETAINE	2,388	2,099	2,070
BIS(2-ETHYLHEXYL)PHTHALATE	ns	2,840	ns
BUTYRYLCARNITINE	0,364	0,813	0,831
CHENODEOXYCHOLIC ACID	ns	8,326	ns
CDP-ETHANOLAMINE	0,822	ns	ns
CIS-ACONITATE-1	0,109	0,666	0,464
CIS-ACONITATE-2	0,388	0,547	0,613
CITRATE	0,101	0,592	0,434
CITRULLINE	1,865	1,324	1,814
CREATINE	1,147	ns	ns
CYCLIC AMP	0,800	0,627	ns
CYCLIC GMP	0,713	ns	ns
CYSTATHIONINE	ns	0,776	ns
CYSTEINE	ns	2,377	ns
DECADIENOYLCARNITINE-2	1,277	ns	ns
DECANOYLCARNITINE	ns	0,858	ns
DECENOYLCARNITINE	0,319	ns	ns
DEOXYADENOSINE MONOPHOSPHATE	0,397	ns	0,600
DEOXYCARNITINE	ns	0,824	ns
DEOXYCHOLATE	ns	8,307	ns
DEOXYCHOLIC ACID	ns	8,256	ns
DEOXYCYTIDINE-1	5,328	3,247	ns
DEOXYCYTIDINE-DIPHOSPHATE	0,619	ns	ns
DEOXYGUANOSINE-MONOPHOSPHATE	0,358	ns	ns
DEOXYURIDINE	ns	1,693	ns
D-GLUCURONOLACTONE	1,767	1,542	ns
DL-2-AMINOCTANOATE	2,209	1,489	ns
DOPAMINE-3	0,491	ns	0,876
D-RIBOSE 5-PHOSPHATE	1,620	1,421	ns
ETHANOLAMINE PHOSPHATE	2,766	2,104	1,600
FRUCTOSE 1,6-BISPHOSPHATE	1,768	2,334	ns
FRUCTOSE 6-PHOSPHATE	2,180	1,410	ns
FUMARATE	0,016	0,176	0,319
GLUCONATE	ns	ns	0,838
GLUCONOLACTONE	2,040	ns	ns
GLUTAMATE	0,164	0,275	0,454
GLUTAMINE	1,700	1,422	ns
GLUTARATE	ns	1,428	1,290
GLUTATHIONE REDUCED	0,806	0,740	ns
GLYCERALDEHYDE 3-PHOSPHATE	0,732	ns	ns
GLYCERATE	0,344	ns	ns
GLYCEROL 3-PHOSPHATE	1,586	ns	1,359
GLYCINE	2,257	1,897	ns
GUANINE-1	ns	1,904	ns

GUANINE-2	1,221	ns	ns
GUANOSINE	ns	1,944	ns
GUANOSINE MONOPHOSPHATE	ns	0,755	ns
HEXANOYLCARNITINE	0,431	ns	ns
HEXOSE	ns	0,498	ns
HISTIDINE	1,225	ns	ns
HOMOCYSTEINE THIOLACTONE	1,388	1,248	ns
HOMOSERINE	1,534	1,288	1,303
HYDROQUINONE	0,643	ns	ns
HYDROXYKYNURENINE	ns	0,770	ns
HYDROXYPHENYLLACTATE	3,376	6,572	2,597
HYPOXANTHINE	6,616	2,141	ns
INDOLEACETALDEHYDE	1,781	4,537	ns
INOSINE	ns	2,392	ns
INOSINE MONOPHOSPHATE	15,874	2,170	ns
ISOCITRATE	0,221	ns	0,528
ISOLEUCINE	1,380	1,349	1,433
ISOVALERYLCARNITINE	0,296	ns	0,508
KYNURENINE	1,266	1,191	1,321
L-ALANINE	0,076	0,057	0,353
L-CARNITINE	0,274	0,719	0,586
LEUCINE	1,466	1,338	ns
L-ORNITHINE	ns	1,416	ns
LYSINE	ns	1,249	ns
MALATE	0,047	0,212	0,393
METHIONINE SULFOXIDE	1,844	1,598	1,373
METHIONINE-1	1,321	1,327	ns
METHIONINE-2	1,394	1,480	ns
METHYLTHIOADENOSINE	1,261	ns	ns
MEVALONATE	ns	0,689	ns
N,N,N-TRIMETHYLLYSINE	2,171	1,406	1,398
N1-ACETYLSPERMINE	2,604	ns	ns
N-A-ACETYL-L-ARGININE	1,772	ns	ns
N-ACETYLALANINE	0,753	ns	1,178
N-ACETYLASPARAGINE	3,386	2,957	1,469
N-ACETYLASPARTATE	0,702	0,720	ns
N-ACETYLASPARTYLGLUTAMATE	0,753	ns	ns
N-ACETYLGLUTAMATE	1,573	1,882	1,477
N-ACETYLGLYCINE	2,255	1,814	1,877
N-ACETYLLEUCINE	ns	ns	1,234
N-ACETYLMETHIONINE	0,833	ns	ns
N-ACETYLNEURAMINATE	0,805	0,492	0,916
N-ACETYLPHENYLALANINE	1,727	ns	ns
N-ACETYLPUTRESCINE	ns	6,001	ns
N-ACETYLSERINE	2,787	2,412	2,387
N-FORMYL-L-METHIONINE	1,247	ns	1,178

NICOTINAMIDE MONONUCLEOTIDE	1,397	ns	ns
N-METHYLGLUTAMATE	0,164	0,396	ns
O-ACETYLCARNITINE	11,372	2,006	2,627
O-ACETYLCARNITINE-2	11,453	2,255	2,183
O-ACETYLSERINE	0,147	0,230	0,410
O-SUCCINYL-HOMOSERINE-1	0,244	0,416	0,252
OXOGLUTARATE	0,075	0,314	0,408
PALMITOYLCARNITINE	ns	1,771	1,390
PANTOTHENATE	ns	ns	1,099
PHENYLALANINE	1,205	1,175	ns
PHENYLETHANOLAMINE	ns	1,198	ns
PHENYLLACTATE	2,165	ns	1,405
PHOSPHOCREATINE	ns	0,419	ns
PHOSPHORYLCHOLINE	1,540	1,274	ns
PHOSPHOSERINE	0,274	0,251	ns
PIPECOLATE	2,576	5,066	2,497
P-OCTOPAMINE	2,455	ns	ns
PORPHOBILINOGEN-2	0,403	0,727	ns
PROLINE	0,647	0,144	ns
PROPIONYLCARNITINE	0,636	ns	0,761
PROPIONYLCHOLINE	ns	0,604	
PTERIN	ns	ns	1,768
PUTRESCINE-NH3	ns	1,299	ns
PYRIDOXAL-PHOSPHATE	1,360		ns
PYRIDOXINE-1	0,761		ns
PYRIDOXINE-2	0,728		ns
PYROGLUTAMATE	0,762	1,319	ns
PYRUVATE	0,448	0,405	ns
RIBOFLAVIN	ns	ns	1,266
S-ADENOSYLHOMOCYSTEINE-1	ns	0,622	ns
SALSOLINOL-1	ns	0,802	ns
SALSOLINOL-2	ns	0,829	ns
SARCOSINE	1,155	ns	ns
SEDOHEPTULOSE-7-PHOSPHATE	4,337	3,558	ns
SPERMIDINE	1,253	1,315	ns
SPERMINE	0,644	ns	0,795
SUBERATE	ns	ns	1,099
SUCCINATE	0,638	0,534	0,729
TAURINE	ns	0,492	ns
THREONINE	1,444	ns	ns
THYMIDINE-MONOPHOSPHATE	0,514	0,609	ns
TRANS-2-OCTENOYL-L-CARNITINE	ns	0,808	ns
TRANS-ACONITATE	0,270	ns	0,579
TRIGONELLINE	0,623	0,331	ns
TRIMETHYLAMINE	0,335	0,745	ns
TRYPTOPHAN	1,438	1,465	1,225

TYRAMINE	2,012	1,303	ns
TYROSINE	1,519	1,477	1,221
URACIL-1	0,492	0,617	ns
URIDINE MONOPHOSPHATE	0,481	0,445	ns
VALINE	2,052	1,366	1,359
XANTHOSINE	ns	1,241	1,360

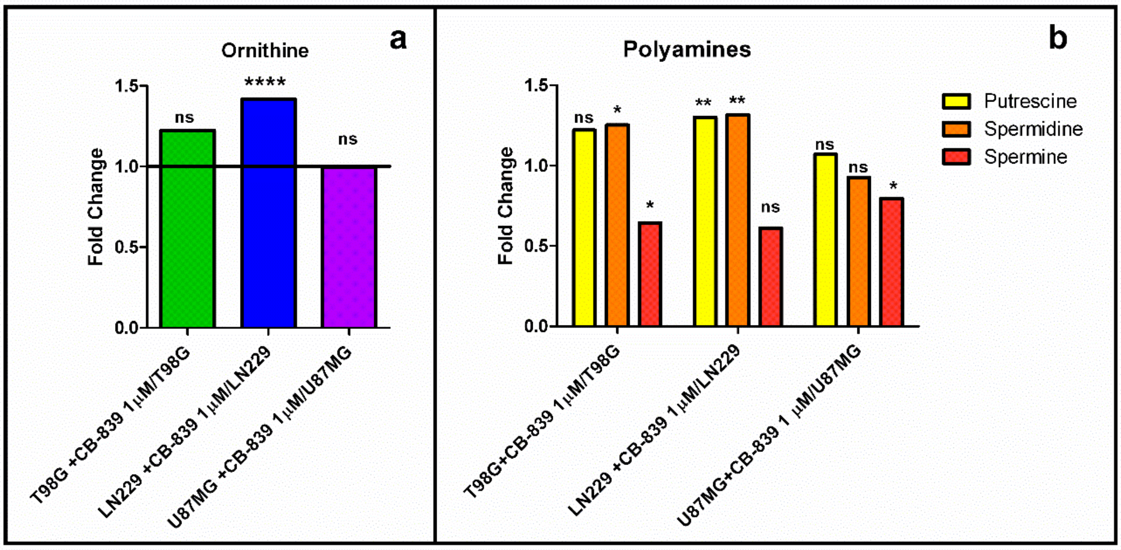

Figure S1. Ornithine (**a**) and polyamines (**b**) levels upon GLS inhibition by CB-839. The samples were run in triplicate. For more details, see "Material and Methods". ns: non-significant.

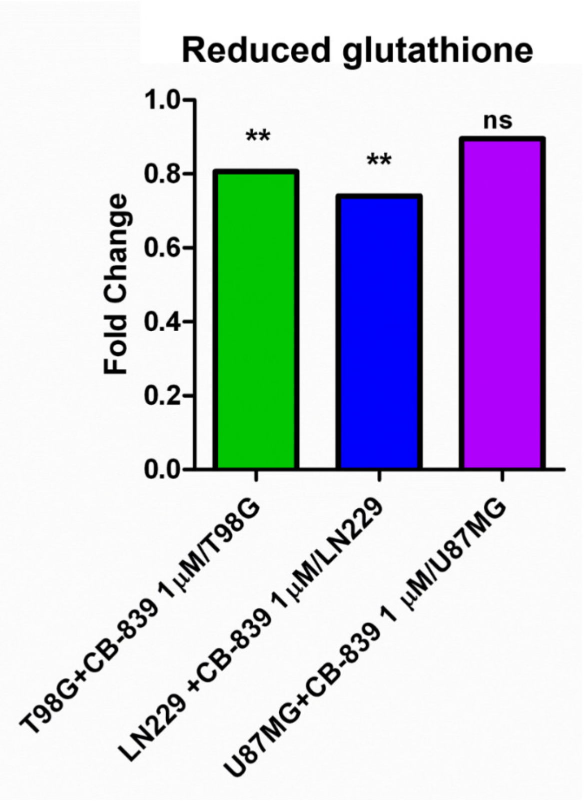

Figure S2. Glutathione levels upon GLS inhibition by CB-839. The samples were run in triplicate. For more details, see "Material and Methods". ns: non-significant.

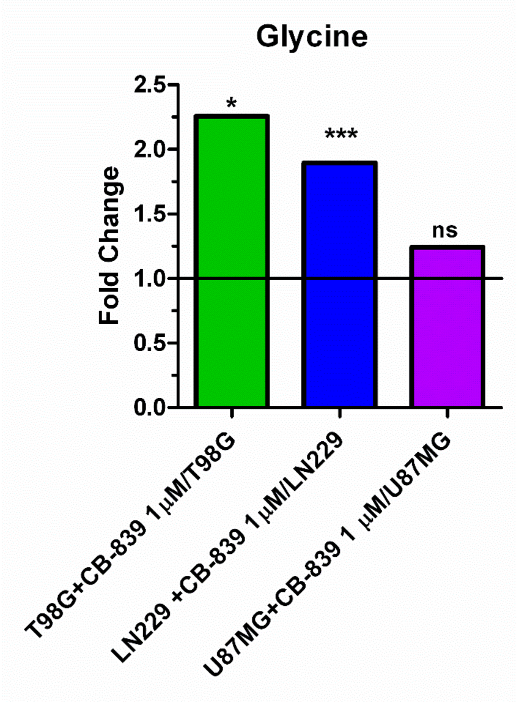

Figure S3. Glycine levels upon GLS inhibition by CB-839. The samples were run in triplicate. For more details, see "Material and Methods". ns: non-significant.

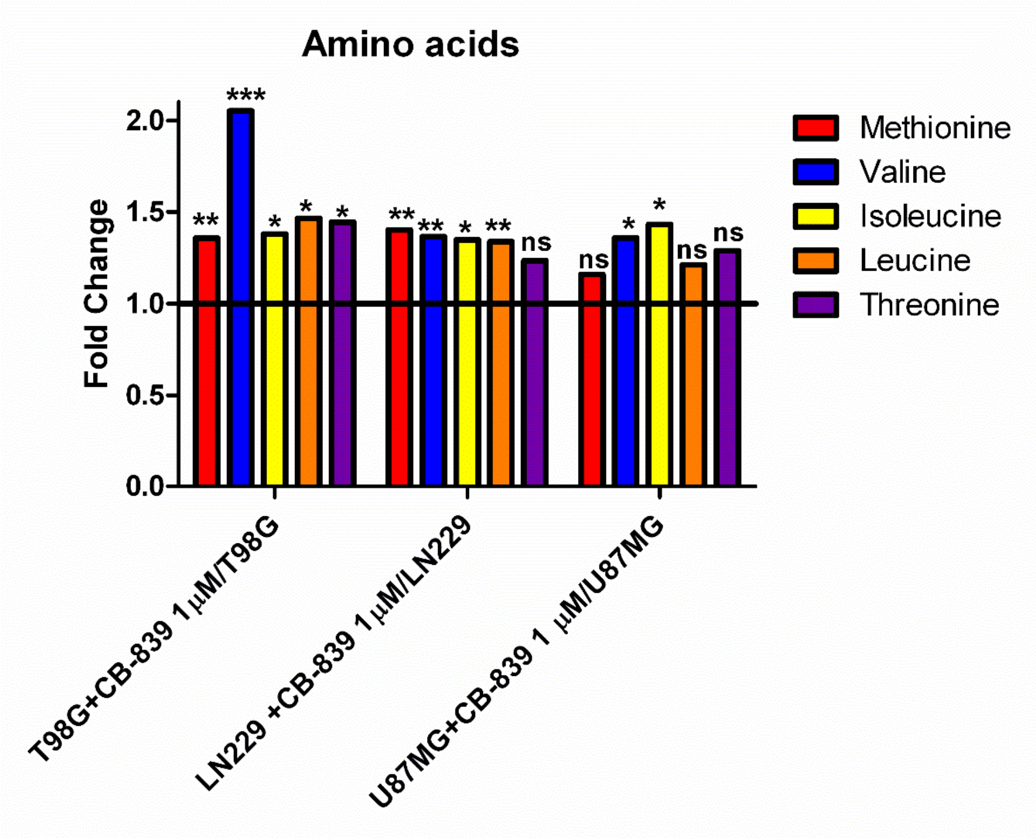

Figure S4. Levels of some amino acids upon GLS inhibition by CB-839. The samples were run in triplicate. For more details, see “Material and Methods”. ns: non-significant.
